# ICTV Virus Taxonomy Profile: *Yueviridae* 2023

**DOI:** 10.1099/jgv.0.001904

**Published:** 2023-10-11

**Authors:** Mart Krupovic, Yuri I. Wolf, Eugene V. Koonin, Jens H. Kuhn

**Affiliations:** ^1^​ Institut Pasteur, Université Paris Cité, Paris 75015, France; ^2^​ National Center for Biotechnology Information, National Library of Medicine, National Institutes of Health, Bethesda, MD 20894, USA; ^3^​ Integrated Research Facility at Fort Detrick, National Institute of Allergy and Infectious Diseases, National Institutes of Health, Fort Detrick, Frederick, MD 21702, USA

**Keywords:** ICTV Report, taxonomy, *Yueviridae*, yuyuevirus

## Abstract

*Yueviridae* is a family of negative-sense RNA viruses with genomes of 7.8–8.2 kb that have been associated with crustaceans, insects, stramenopiles and plants. The yuevirid genome consist of two segments, each with at least one ORF. The large (L) segment ORF encodes a large protein containing an RNA-directed RNA polymerase domain. The small (S) segment ORF encodes a nucleocapsid protein. This is a summary of the International Committee on Taxonomy of Viruses (ICTV) Report on the family *Yueviridae*, which is available at http://www.ictv.global/report/yueviridae.

## Abbreviation

L, large; N, nucleocapsid; RdRP, RNA-directed RNA polymerase; S, small.

## Virion

Unknown.

## Genome

**Fig. 1.**
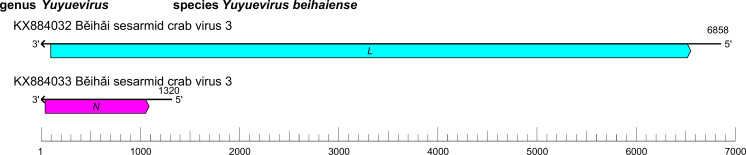
Genome organisation Běihǎi sesarmid crab virus 3. ORFs are coloured according to the predicted protein function [*L*, large protein gene encoding an RNA-directed RNA polymerase (RdRP) domain; *N,* gene encoding a nucleocapsid protein].

The yuevirid genome comprises two segments of linear negative-sense RNA with a total length of 7.8–8.2 kb (large (L) segment, 6.6–6.9 kb; small (S) segment, 1.2–1.3 kb) [1–5] (Table 1, Fig. 1). The L segment ORF (*L*) encodes an RNA-directed RNA polymerase (RdRP) with a GDP : polyribonucleotidyltransferase (PRNTase) domain that is related to that of viruses in the order *Mononegavirales*, suggesting a similar capping mechanism. The S segment ORF (*N*) encodes a nucleocapsid protein structurally related to the homologues encoded by mononegavirals, in particular paramyxovirids.

**Table 1.** Characteristics of members of the family *Yueviridae*

Example	Běihǎi sesarmid crab virus 3 (L, KX884032; S, KX884033), species *Yuyuevirus beihaiense,* genus *Yuyuevirus*
Virion	Unknown
Genome	7.8–8.2 kb of bisegmented negative-sense RNA
Replication	Unknown
Translation	Unknown
Host range	Branchiopod, decapod and isopod crustaceans, dipteran and thysanopteran insects, lycopodiopsid and polypodiopsid plants and various macrophytes, and stramenopiles
Taxonomy	Realm *Riboviria*, kingdom *Orthornavirae*, phylum *Negarnaviricota*, class *Yunchangviricetes*, order *Goujianvirales*; the family includes the genus *Yuyuevirus* and two species

## Replication

Unknown.

## Taxonomy

**Fig. 2.**
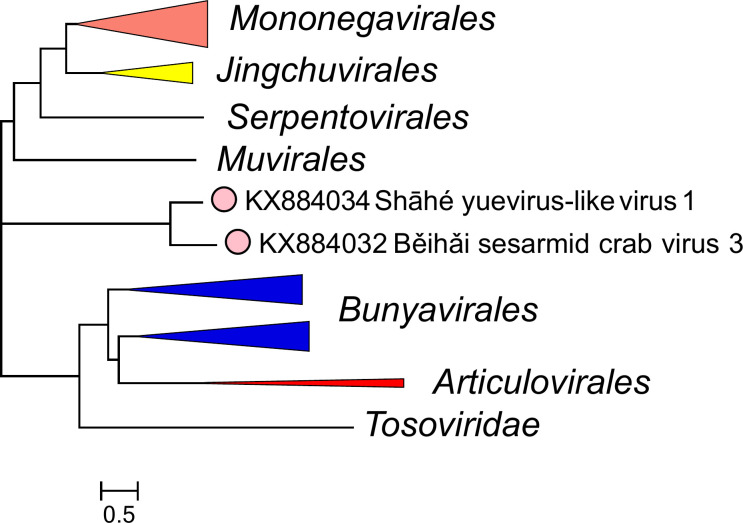
Phylogenetic relationships of yuevirids. A phylogenetic tree was reconstructed for an alignment of the RdRP core domains of the ICTV Virus Metadata Resource (VMR) set of *Negarnaviricota* using the FastTree program. Branches for viruses other than yuevirids are collapsed according to order taxon; the complete tree is available in the full *Yueviridae* ICTV Report. Scale: substitutions/site.

Current taxonomy: ictv.global/taxonomy. The family *Yueviridae* includes at least one genus and two species for the viruses Běihǎi sesarmid crab virus 3 and Shāhé yuevirus-like virus 1, found in unspecified sesarmid crabs (Crustacea: Decapoda) and freshwater isopods (Crustacea: Isopoda), respectively, in China [1] (Fig. 2). Unclassified yuevirids have been associated with diverse crustaceans and insects [6], stramenopiles [7–9], tracheophytes [3], and unspecified macrophytes [5]. Thus, the diversity of yuevirids is likely vastly underestimated [2, 4].

## Resources

The full ICTV Report on the family *Yueviridae* is available at: ictv.global/report/yueviridae.
